# Effects of peer-led education on knowledge, attitudes, practices of stoma care, and quality of life in bladder cancer patients after permanent ostomy

**DOI:** 10.3389/fmed.2024.1431373

**Published:** 2024-10-18

**Authors:** Juan-Ying Ding, Ting-Ting Pan, Xu-Jing Lu, Xiao-Ming You, Jia-Xian Qi

**Affiliations:** ^1^The First People’s Hospital of Linping, Hangzhou, China; ^2^Second Affiliated Hospital, School of Medicine, Zhejiang University, Hangzhou, China

**Keywords:** bladder cancer, ostomy, stoma care, peer-led intervention, knowledge, attitudes, and practices, quality of life

## Abstract

**Objective:**

To investigate the effects of peer-led intervention on knowledge, attitudes, and practices (KAP) of stoma care, as well as quality of life in bladder cancer patients post-permanent ostomy.

**Methods:**

A series of 340 eligible bladder cancer patients who underwent permanent ostomy from January 2019 to December 2022 were enrolled in this study. These participants were randomly assigned to the intervention group (peer-led intervention) and the control group (routine health intervention) using random number table, with 170 cases in each group. A 30-item questionnaire was used to evaluate knowledge, healthy attitudes, and healthy practices (KAP) of disease; the WHO Quality of Life-100 (WHOQOL-100) was utilized to assess the quality of life among patients; and the incidence of complications in two groups were also recorded during six-month intervention. For the comparison of continuous variables within and between groups, paired sample and independent *t*-test were applied. The categorical variables analyzed using *x^2^* test or rank-sum test.

**Results:**

After six-month intervention, 144 participants in the intervention group and 151 participants in the control group were finally retained in this study. The scores of the 20 items in KAP (including basic knowledge of disease, basic knowledge of ostomy, observation of stoma, etc.) in the intervention group were significantly higher than those in the control group (all *p* < 0.05); the scores of 12 items in WHOQOL-100 (including the positive feelings, thinking, learning, memory and concentration, etc.) in the intervention group were markedly higher than those in the control group, while negative feelings and dependence on medical support in the intervention group were significantly lower than those in the control group (all *p* < 0.05); the total rate of complications in the intervention group was significantly lower than that in the control group (18.31% vs. 31.13%, *p* < 0.05).

**Conclusion:**

The peer-led intervention has a positive effect on improving patients’ KAP of stoma care and quality of life and reducing the rate of complications, which enables it to be a favorable intervention approach for patients with permanent ostomy.

## 1 Introduction

Bladder cancer, as a common urinary cancerous disease, poses a serious threat on personal health and family well-being, as well as social healthcare burden (1). In the United Nations alone, the number of newly diagnosed as bladder cancer has reached 83,730 and 17,200 died from it in 2021 (2). For these bladder cancer patients with muscle invasion or high-risk metastasis, urinary ostomy is a routinely effective procedure after radical cystotomy. Patients with permanent ostomy are prone to experience negative psychological, physiological, and social relationship changes after operation, which may result in subsequent barriers to stoma management and patient’s daily life (3). According to the results of several studies, patients’ knowledge, attitudes, and practices (KAP) about disease play a vital role in the management of chronic diseases, and their self-care ability and quality of life can be improved through enhanced KAP education and intervention (4–6). Based on these findings mentioned above, we suppose that KAP may have a potential role to ease stoma self-care challenges faced by bladder cancer patients undergoing home care or out-patient intervention after permanent ostomy.

Peer-led intervention is a validated health promotion strategy that involves sharing concepts, experiences, and other information among peers with similar experiences or conditions, and its core lies in effective communication between peer educators and recipients, leading to the improvements in cognitions and behaviors among educators and recipients (7, 8). Over the past decade, peer-led intervention has been widely applied in a variety of settings and populations, and it has been proved to be practical way of promoting individual’s KAP and pursuing health goals in previous literature (9–11). With modern communication methods advancing, including instant messaging, online interviews, and video conferences, peer-led intervention is becoming a convenient, efficient, and popular intervention approach (12). Peer-led intervention has been reported to have a good clinical performance in many studies on the management of chronic diseases such as diabetes, rheumatoid arthritis, and cancerous diseases (13–15). However, it still lacks reports regarding peer-led intervention on KAP of stoma care and quality of life for bladder cancer patients in the present literature, especially for permanent ostomy patients after urinary diversion. In order to fill this knowledge gap, we conducted a case–control study to investigate the feasibility and effectiveness of peer-led intervention on KAP and quality of life in bladder cancer patients after permanent ostomy.

## 2 Patients and methods

### 2.1 Patients

**Figure 1:**
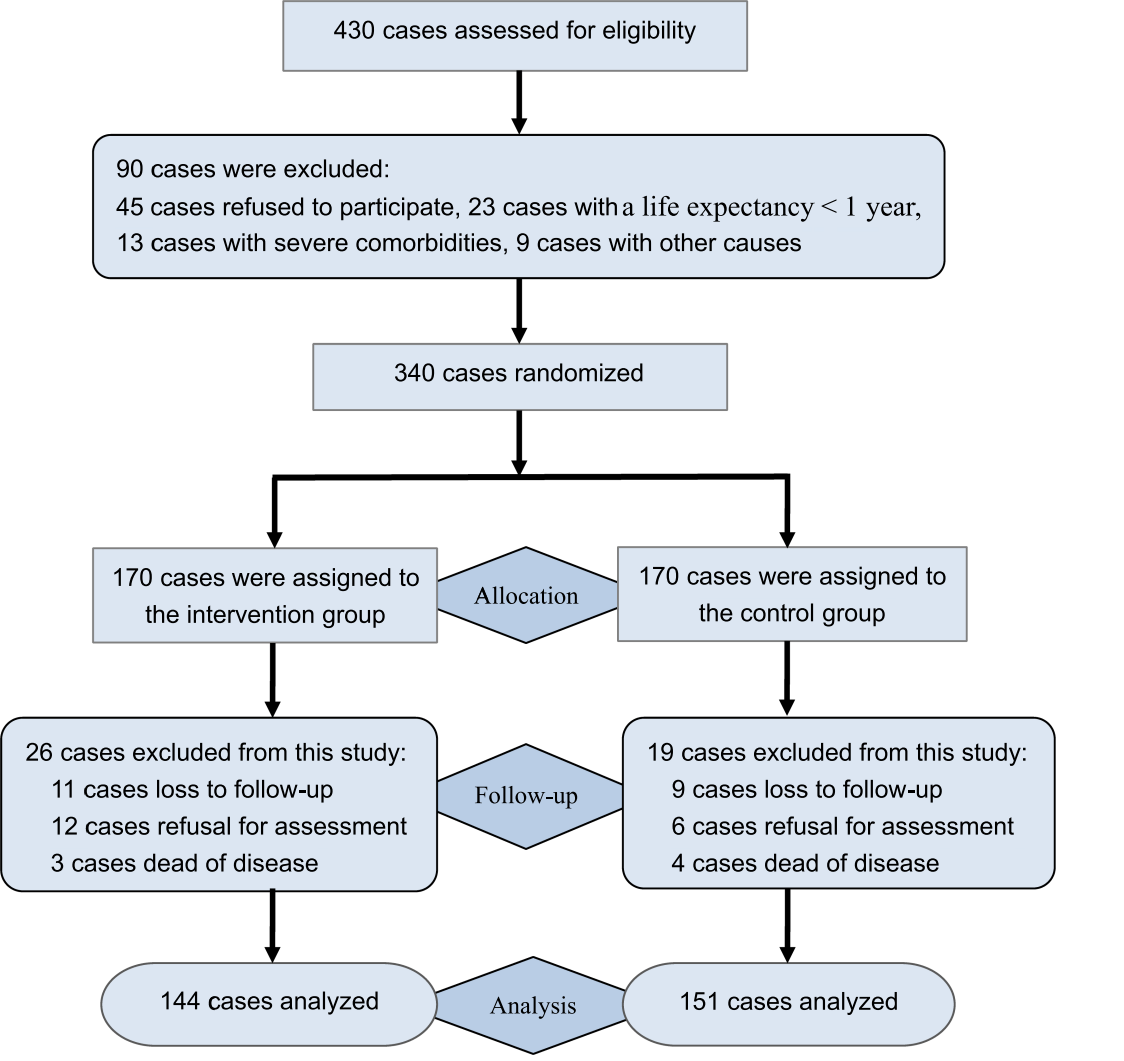
Flowchart of the study.

The study participants consisted of bladder cancer patients who underwent permanent ostomy in Hangzhou District from January 2019 to December 2022, and these participants were recruited through posters and snowball methods. This study was approved by the Medical Ethics Committee of Linping Hospital (Approval no: 2019–015). A preliminary test was used to calculate the sample size referring to the previous study (16). The *α* value was set at 5% (two-tailed test), the power was set at 80% (1-*β*), and the effect size was set at 0.15. The *σ* represents the evaluated value of the standard deviation in quality of life between two groups. It showed that the target sample size should be 236 cases. Given potential dropout or follow-up loss with a 30% attrition rate, the target sample size was set at ≥338 cases. Prior to the inclusion, all patients were screened for study eligibility. Inclusion criteria of this study were set as follows: confirmed bladder cancer and underwent permanent ostomy; healthy condition with a life expectancy of more than 1 year; voluntarily participating in this study and giving informed consent. Exclusion criteria included: patients with temporary ostomy; dropped out or lost to follow-up; severe comorbidities or communication disorders. During the course of recruitment, a total of 430 patients were enrolled to be screened, and 340 patients who met eligible criteria were randomized into the intervention group and the control group. A random number sequence was generated through a random number table with the help of a statistician, and random numbers were distributed to each patient to achieve random grouping in a 1:1 ratio. To avoid allocation bias, randomization was conducted by separate researchers who were not involved in the conception and data analysis of this study. Of them, 170 participants were allocated to the intervention group, receiving peer-led intervention, while another 170 participants were assigned to the control group, receiving routine health intervention. After six-month intervention, 144 participants were included in the intervention group, and 26 participants were excluded from this group due to loss to follow-up, refusal for assessment, or dead of disease; 151 participants retained in the control group, while 19 cases excluded from this group due to the above-mentioned causes. Finally, a total of 295 cases completed the 6-month intervention and were included in data curation. The flowchart of this study was described in Figure 1.

### 2.2 Methods

Participants in both groups were followed-up for 6 months by WeChat (a social communication medium), telephone, or out-patient visit. During this period, the control group received routine health intervention, including basic disease knowledge, stoma care, postoperative rehabilitation, and other stoma-related concerns. On the basis of the control group, the intervention group received peer-led intervention. Thirty-four volunteers who have full willingness, good communication skills, and stoma care experiences over 1 year were recruited as peer educators through posters. The protocols of training were similar to the previous study (17), consisting of 2 weeks of training sessions to strengthen their knowledge of disease and stoma, diet and exercise, complication observation and management, and other related self-care skills. Furthermore, problem-solving methods and sharing tips are also trained, such as how do you handle pouch leakage; if you handle it well yourself, how do you share your successful experiences with other patients? In brief, the training course focuses on knowledge and skills acquisition related to healthy concept and behavior, effective facilitation, and group management. After the training course 34 educators were assigned to 17 peer teams by random number table. Then, 12 members including 2 educators and 10 participants constituted a peer-led team, and these members were invited to a WeChat communication group. In the initial month, weekly video conferences, online or in-person interviews were conducted to alleviate the sense of unfamiliarity and communicate disease knowledge, self-care skills, and handling experiences. In the subsequent months, educators were responsible for reporting to medical staff regarding the status of stoma care, psychological aspects, and interventing effectiveness of their members. In this study, blinding of participants and study staff failed to apply owing to the nature of the intervention, but the raw data collectors and statistician were blinded to data analysis, making this project an open blinded study.

### 2.3 Evaluating variables

Baseline data (age, gender, education level, family income, and others) were collected at the entry of this study. A 30-item questionnaire was used to evaluate participant’s KAP status, including 12 items about stoma knowledge (basic knowledge of disease, basic knowledge of ostomy, observation of stoma, method of pouch replacement, emptying the pouch, etc.), 9 items concerning healthy attitudes (optimistic mentality to disease, optimistic mentality to stoma, trust in medical staff, trust in peer educators, willing to correct bad habits, etc.), and 9 items of healthy practices (maintaining healthy diet, maintaining healthy behaviors, learning relevant books, learning relevant videos, skilled in pouch replacement, etc.) (18). The result of each item was graded on a five-point Likert scale system with indication from very poor to very good. The assessment of quality of life adopted WHO Quality of Life-100 (WHOQOL-100), which involved six domains (physical, psychological, independence, social relationship, environment, and spirituality) and 24 items by using the same Likert scale system (19). Overall, a higher score indicates a better outcome in quality of life. Complications such as dermatitis, stoma infection, local necrosis, mucocutaneous separation, stoma stricture, and others in two groups were recorded during the intervention.

### 2.4 Statistical analysis

The data were analyzed by SPSS25.0 software. The continuous variables were presented as mean ± standard deviation (*x* ± *s*). Normality was assessed using Shapiro–Wilk test. Independent *t*-test was used to compare the values between the two groups, and paired *t*-test was utilized to compare the changes between pre-intervention and post-intervention within an individual group. The categorical variables were expressed as frequency and percentages (%), analyzed by *x^2^* test, and the ranked data were analyzed by the rank-sum test. *p* < 0.05 indicates that the difference was statistically significant.

## 3 Results

### 3.1 Comparison of baseline data

Before the intervention, there was no significant difference in gender, age, education level, marital status, family income, comorbidities, and surgical type between two groups (all *p* > 0.05). As shown in Table 1.

**Table 1:** Comparison of baseline data between two groups [*n*(%)].

Characteristics	Intervention group (144)	Control group (151)	*χ*2*/Z*	*P*
Gender				
Male	108(75.00)	106(70.20)	0.853	0.365
Female	36(25.00)	45(29.80)		
Age (years)				
<45	17(11.81)	22(14.57)		
45 ~ 60	46(31.94)	62(41.06)	4.172	0.124
>60	81(56.25)	67(44.37)		
Education level				
Elementary	22(15.28)	29(20.14)		
Junior/Senior high	78(54.17)	74(49.01)	1.074	0.584
College/University	44(30.56)	48(31.79)		
Marital status				
Married	92(63.89)	84(55.63)	2.329	0.507
Unmarried	8(5.56)	10(6.62)		
Divorced	23(15.97)	27(17.88)		
Widowed	21(14.58)	30(19.87)		
Family income				
(CNY/month) < 10,000	33(22.92)	31(20.53)	1.276	0.735
10,000 ~ 20,000	72(50.00)	77(50.99)		
>20,000	39(27.08)	43(28.48)		
Comorbidities				
Hypertension	49(34.03)	48(31.79)	0.168	0.682
Diabetes	27(18.75)	31(20.53)	0.148	0.701
Coronary heart disease	26(18.06)	33(21.85)	0.668	0.415
Others	22(15.28)	29(19.21)	0.795	0.373
Surgical type				
Ileal conduit	76(52.78)	85(56.29)	0.858	0.651
Bilateral ureterostomy	53(36.81)	48(31.79)		
Others	15(10.42)	18(11.92)		

### 3.2 Comparison of KAP

There was no statistically significant difference in the score of each item of KAP between two groups before intervention (*p* > 0.05). At 6 months after intervention, the scores of 7 items in knowledge dimension such as basic knowledge of disease, basic knowledge of ostomy, observation of stoma, use of ancillary devices, handling pouch leakage, peristomal skin care, and complication management of the intervention group were significantly higher than those of the control group; the scores of 6 items in attitudes dimension including optimistic mentality to disease, optimistic mentality to stoma, trust in peer educators, willing to correct bad habits, willing to help others, and willing to correct bad emotion were significantly higher than those of the control group; the scores of 7 items in practices dimension including maintaining healthy diet, maintaining healthy behaviors, learning relevant books, learning relevant videos, skilled in pouch replacement, experienced in stoma care, and communicated with others of the intervention group were markedly superior to the control group. These differences of aforementioned 20 items were statistically significant (all *p* < 0.05). As shown in Table 2.

**Table 2:** Comparison of KAP between two groups (*x* ± *s*).

Items	Before intervention				After intervention			
	Intervention control group (144) group (151)		*t*	*P*	Intervention group (144)	Control group (151)	*t*	*P*
Knowledge								
Basic knowledge of disease	2.61 ± 0.75	2.52 ± 0.79	1.003	0.317	3.94 ± 0.80	3.47 ± 0.91	4.703	<0.001
Basic knowledge of ostomy	2.89 ± 0.82	2.94 ± 0.76	0.551	0.582	3.91 ± 0.82	3.58 ± 0.87	3.349	0.001
Observation of stoma	2.28 ± 0.60	2.31 ± 0.65	0.411	0.681	4.01 ± 0.49	3.52 ± 0.53	8.235	<0.001
Method of pouch replacement	3.04 ± 0.68	3.17 ± 0.75	1.557	0.120	3.67 ± 0.64	3.58 ± 0.72	1.133	0.258
Emptying the pouch	3.13 ± 0.72	3.06 ± 0.71	0.841	0.401	4.10 ± 0.49	4.16 ± 0.52	1.019	0.309
Measurement of stoma size	3.17 ± 0.84	3.30 ± 0.78	1.378	0.169	3.74 ± 0.89	3.79 ± 0.92	0.474	0.636
Connecting/shutting of pouch	3.15 ± 1.02	2.94 ± 0.96	1.822	0.070	3.95 ± 0.81	3.81 ± 0.70	1.591	0.113
Use of ancillary devices	2.09 ± 0.51	2.14 ± 0.64	0.744	0.481	4.07 ± 0.63	3.65 ± 0.64	5.677	<0.001
Handling pouch leakage	2.37 ± 0.76	2.49 ± 0.80	1.320	0.188	3.56 ± 0.72	3.18 ± 0.83	4.192	<0.001
Peristomal skin care	2.62 ± 0.65	2.53 ± 0.69	1.152	0.250	4.02 ± 0.51	3.83 ± 0.57	3.012	0.003
Purchase and storage of pouch	2.78 ± 0.87	2.89 ± 0.92	1.054	0.293	3.84 ± 0.89	3.68 ± 0.81	1.616	0.107
Complication management	2.01 ± 0.54	1.95 ± 0.57	0.927	0.355	3.32 ± 0.85	2.99 ± 0.89	3.254	0.001
Attitudes								
Optimistic mentality to disease	1.84 ± 0.67	2.00 ± 0.75	1.929	0.055	4.02 ± 0.70	3.14 ± 0.93	9.209	<0.001
Optimistic mentality to stoma	2.74 ± 0.84	2.63 ± 0.78	1.166	0.245	3.27 ± 0.62	2.98 ± 0.84	3.384	0.012
Trust in medical staff	3.37 ± 0.75	3.34 ± 0.71	0.353	0.724	3.85 ± 0.90	3.67 ± 0.86	1.757	0.080
Trust in peer educators	2.56 ± 0.79	2.68 ± 0.84	1.263	0.208	3.96 ± 0.61	3.59 ± 0.78	4.550	0.003
Willing to correct bad habits	3.14 ± 0.67	3.02 ± 0.64	1.573	0.117	3.70 ± 0.82	3.45 ± 0.90	2.490	0.013
Willing to help others	2.45 ± 0.78	2.37 ± 0.76	0.892	0.373	4.08 ± 0.65	3.29 ± 0.80	9.328	<0.001
Willing to correct bad emotion	1.82 ± 0.59	1.78 ± 0.57	0.592	0.554	3.62 ± 0.93	3.28 ± 0.91	3.173	0.002
Confidence in healthy behaviors	2.73 ± 0.65	2.80 ± 0.63	0.939	0.348	3.81 ± 0.64	3.92 ± 0.69	1.418	0.157
Confidence in rehabilitation plan	3.21 ± 0.83	3.14 ± 0.78	0.747	0.456	3.93 ± 0.70	3.87 ± 0.84	0.668	0.526
Practices								
Maintaining healthy diet	2.74 ± 0.79	2.59 ± 0.83	1.588	0.113	3.83 ± 0.85	3.57 ± 0.82	2.674	0.008
Maintaining healthy behaviors	2.67 ± 0.62	2.56 ± 0.70	1.426	0.155	3.92 ± 0.73	3.63 ± 0.77	3.316	0.001
Learning relevant books	1.95 ± 0.61	1.88 ± 0.57	1.019	0.309	2.66 ± 0.79	2.21 ± 0.78	4.922	<0.001
Learning relevant videos	2.21 ± 0.45	2.27 ± 0.52	1.058	0.291	3.71 ± 0.92	3.36 ± 0.96	3.194	0.002
Skilled in pouch replacement	2.26 ± 0.69	2.36 ± 0.68	1.254	0.211	3.82 ± 0.71	3.54 ± 0.75	3.290	0.001
Experienced in stoma care	2.28 ± 0.47	2.35 ± 0.41	1.354	0.173	3.93 ± 0.80	3.69 ± 0.62	2.871	0.024
Communicated with others	2.02 ± 0.63	2.11 ± 0.69	1.168	0.244	3.54 ± 0.88	2.92 ± 0.84	6.191	<0.001
Following doctor’s advice	3.15 ± 0.80	3.04 ± 0.73	1.235	0.218	3.72 ± 0.91	3.85 ± 0.83	1.283	0.201
Regular return visit	–	–	–	–	3.80 ± 0.54	3.94 ± 0.65	2.016	0.084

### 3.3 Comparison of quality of life

There was no significant difference in WHOQOL-100 score between two groups before intervention (*p* > 0.05). After six-month intervention, significant differences in the scores of 14 items of WHOQOL-100 were observed between two groups. Among these items, scores of the positive feelings, thinking, learning, memory and concentration, self-esteem, mobility, activities of daily living, work capacity, personal relationship, social support, healthy and social care, new information and skills, spirituality/religion/personal belief, quality of life from the viewpoint in the intervention group were significantly higher than those in the control group, while scores of negative feelings and dependence on medical support in the intervention group were markedly lower than those in the control group. All differences mentioned above were statistically significant (all *p* < 0.05). As shown in Table 3.

**Table 3:** Comparison of quality of life between two groups (*x* ± *s*).

Items	Before intervention				After intervention			
	Intervention group (144)	Control group (151)	*t*	*P*	Intervention Control group (144) group (151)		*t*	*P*
Physical								
Pain and discomfort	15.14 ± 3.75	14.67 ± 3.82	1.066	0.287	13.86 ± 3.57	13.70 ± 3.38	0.395	0.693
Energy and fatigue	12.75 ± 4.21	13.56 ± 3.79	1.738	0.083	14.37 ± 3.23	13.91 ± 3.35	0.920	0.358
Sleep and rest	13.02 ± 3.50	12.55 ± 3.36	1.177	0.240	13.58 ± 4.15	12.86 ± 4.34	1.455	0.147
Psychological								
Positive feelings	10.11 ± 3.49	9.97 ± 3.75	0.332	0.740	14.39 ± 3.74	13.28 ± 3.86	2.507	0.013
Thinking, learning, memory, and concentration	13.07 ± 3.16	12.89 ± 2.72	0.525	0.600	14.34 ± 3.22	13.25 ± 2.97	3.024	0.003
Self-esteem	12.30 ± 3.17	11.91 ± 3.78	0.962	0.368	15.12 ± 3.59	12.53 ± 4.11	5.753	<0.001
Body image and appearance	9.42 ± 3.19	8.73 ± 3.06	1.896	0.059	13.47 ± 3.61	12.89 ± 3.98	1.309	0.192
Negative feelings	13.45 ± 4.31	14.24 ± 3.85	1.662	0.098	11.53 ± 3.86	13.28 ± 3.50	4.083	<0.001
Independence								
Mobility	11.61 ± 3.60	11.52 ± 3.91	0.205	0.837	13.82 ± 3.86	12.36 ± 4.24	3.088	0.002
Activities of daily living	11.83 ± 3.52	12.26 ± 3.80	1.007	0.315	14.09 ± 2.97	13.05 ± 3.45	2.769	0.006
Dependence on medical support	14.67 ± 3.14	15.13 ± 2.94	1.299	0.195	12.27 ± 4.31	13.52 ± 3.79	2.648	0.009
Work capacity	10.59 ± 3.48	10.67 ± 3.46	0.198	0.843	13.40 ± 2.84	12.11 ± 3.23	3.636	<0.001
Social relationship								
Personal relationship	13.14 ± 3.59	12.47 ± 3.12	1.713	0.088	15.84 ± 3.02	14.73 ± 2.87	3.237	0.001
Social support	12.37 ± 3.61	12.72 ± 4.28	0.760	0.472	15.96 ± 3.47	14.54 ± 3.38	3.560	<0.001
Sexual activity	12.01 ± 3.10	12.33 ± 3.65	0.813	0.443	10.08 ± 2.75	9.76 ± 3.20	0.919	0.359
Environment								
Physical safety and security	12.08 ± 3.48	12.45 ± 3.23	0.947	0.344	11.79 ± 3.56	12.62 ± 4.09	1.855	0.065
Home environment	14.10 ± 3.76	13.51 ± 3.84	1.333	0.184	13.15 ± 3.84	13.87 ± 3.56	1.671	0.096
Financial resources	12.46 ± 3.75	12.89 ± 4.25	0.920	0.358	10.84 ± 3.37	11.17 ± 4.14	0.752	0.476
Health and social care	10.31 ± 3.82	11.08 ± 3.98	1.694	0.091	13.83 ± 2.84	12.69 ± 3.07	3.307	0.001
New information and skills	11.62 ± 2.83	11.94 ± 3.41	0.879	0.409	14.81 ± 3.25	11.34 ± 3.28	9.123	<0.001
Recreation and leisure	10.94 ± 2.88	11.17 ± 2.90	0.683	0.495	12.32 ± 3.42	11.95 ± 2.85	1.007	0.347
Physical environment	11.53 ± 3.27	12.15 ± 3.65	1.534	0.126	12.80 ± 4.03	12.43 ± 3.72	0.820	0.413
Transportation	11.59 ± 2.84	11.26 ± 3.29	0.920	0.358	11.76 ± 3.18	11.07 ± 3.11	1.884	0.061
Spirituality								
Spirituality/religion/personal belief	10.08 ± 2.93	9.62 ± 3.17	1.293	0.197	14.69 ± 3.74	13.30 ± 3.86	3.139	0.002
Quality of life from the viewpoint	11.02 ± 3.37	10.84 ± 2.81	0.429	0.668	14.62 ± 3.61	12.48 ± 3.49	5.177	<0.001

### 3.4 Comparison of complications

During the 6-month intervention, 26 complications occurred in 22 participants of the intervention group, accounting for 18.31% (26/144) in participants, while 47 complications occurred in 42 participants of the control group, accounting for 31.13% (47/151) in participants. The total rate of complication in the intervention group was significantly lower than that in the control group, and differences were statistically significant (*x^2^* = 6.425, *p* = 0.011). As shown in Table 4.

**Table 4:** Comparison of complications between the two groups [*n*(%)].

Complications	Intervention group (144)	Control group (151)	*χ*2	*P*
Local dermatitis	6 (4.17)	9 (5.96)		
Stoma infection	4 (2.78)	7 (4.64)		
Local necrosis	3 (2.08)	6 (3.97)		
Mucocutaneous separation	3(2.08)	6 (3.97)		
Stoma stricture	2(1.39)	5 (3.31)		
Retraction	1(0.69)	3 (1.99)		
Prolapse	0 (0.00)	4 (2.65)		
Granulomatosis	2 (1.39)	0 (0.00)		
Fistula	3 (2.08)	2 (1.32)		
Others	2 (1.39)	5 (3.31)		
Total rate^*^	26 (18.31)	47 (31.13)	6.425	0.011

^*^There are 2 cases with 2 complications and 1 cases with 3 complications in the intervention group, and 3 cases with 2 complications and 2 cases with 3 complications in the control group.

## 4 Discussion

Adapting to carrying an ostomy pouch for life is often an additional burden for bladder cancer patients after permanent ostomy. Most of patients will struggle to maintain long-term self-management under the enormous stress and anxiety caused by disease and ostomy. Therefore, seeking an appropriate educational intervention plays an important role in the improvements of postoperative rehabilitation, stoma management, and quality of life, especially for those patients with home care or lacking professional care from medical institutions. Peer-led intervention has been proven to be an effective intervention program for health promotion in various fields since it was proposed in 1987 (20). Poudel et al. (21) considered that peer-led cancer education program was a beneficial way to encourage participants in active learning and participate in problem-solving and self-reflection sustainably. Yip et al. (22) concluded that peer-led nutrition education had a positive effect on the dietary health of school-age adolescent. Furthermore, there was an agreement that patients’ knowledge can directly link to their practices while the impacts of knowledge on practices can also be enhanced or jeopardized by their attitudes, and the educational intervention based on the KAP model has also been demonstrated as an effective way to long-term self-management of patients with chronic disease (23–25). In this study, we tested this viewpoint and confirmed the effectiveness of peer-led intervention on KAP of stoma care, quality of life, and stoma complications in patients with permanent ostomy.

In the assessment of patients’ KAP, we found that the scores of 7 items in knowledge, 6 items in attitudes, and 7 items in practices of the intervention group were notably higher than those of the control group. This implies that the peer-led intervention can shift patients’ KAP on stoma care from negative to positive more effectively compared with the routine health intervention. The main reasons of which probably includes (26, 27): First, the educators have a physiological and psychological situations as same/similar as the patients’, easier to develop a good connection and effective communication with peers, give them better social support, and further alleviate patients’ alienation, frustration, and other negative emotions; Second, each educator shares his/her experience of stoma care with the rest members of peer-led team by sharing and demonstrating detail skills, so as to help patients to improve their self-care ability; Third, knowledge and attitudes are the foundation of practices while the improvement of cognition and attitudes can further promote healthy behaviors. For example, when a participant has learned the relevant knowledge with others’ help, he/she successfully handles a problem related to stoma care, and the participant possibly has an optimistic attitude toward the disease and stoma care. After that, he/she is more likely to help others in peer-led team, thereby constituting a mutual optimistic influence on each other (28). It will offer a further facilitation to promote the level of practices in some way.

The WHOQOL-100 is an international scale developed by the World Health Organization to measure individuals’ health-related quality of life, and it has been widely used in different countries with multiple language versions over the past decades, making the outcomes of quality of life more comparable across different cultural backgrounds (29, 30). This scale has good properties such as reliability, validity and responsiveness with multidimensional perspectives, including the aspects of physical health, psychological health, level of independence, state of social relationship, environmental factors, and personal beliefs for life (31). Also, the use of WHOQOL-100 may serve as a reliable feedback on the effectiveness of peer-led intervention in the assessment of quality of life. In this study, our results showed that 14 items of WHOQOL-100 in the intervention group were significantly higher than those of the control group, suggesting that the peer-led intervention can obviously improve the quality of life of participants. However, most of items were seen in physical, psychological, independence and social relationship domains, and only two items were found in the environment domain, indicating that the peer-led intervention had a greater impact on participants’ physical, psychological, and social aspects while less on environment domain. The reason may be that environment factors are difficult to make a noticeable improvement in a few months. Based on the findings of previous studies, postoperative improvement or reduction of quality of life can reflect patients’ adaption to their life with ostomy (32, 33). In this study, our results revealed that the peer-led intervention presented a substantial improvement of patients’ adaption to stoma, which was also consistent with other’s study (34).

A previous report on ostomy patients pointed out that the educational intervention can decrease the rate of complications (35), which was also found in our study. During six-month intervention, the total rate of complications in the intervention group was obviously lower than that in the control group (18.31% vs. 31.13%). As others’ reports, most of stoma patients sought for help from professional guidance in stoma care, especially for patients with permanent ostomy, and those patients can benefit from more educational intervention and reduce the rate of complications through mutual support (35–37). By considering the above viewpoints and our findings, the conclusion can be drawn that peer-led intervention is a feasible and effective approach for bladder cancer patients after permanent ostomy. Peer support is understood as a form of mutual support between individuals and educators with similar experiences, which is distinct from professional support (38). As previously reported, the promotion of patients’ KAP may be the fundamental mechanism by which peer-led education improves the outcomes of individuals in various diseases (9, 39). In our opinion, by setting an example, patients’ desire for well-being can be motivated and enhanced. With the help of experienced educators, gaps in medical services outside of healthcare institutions have been filled, thereby improving patients’ KAP and quality of life, and reducing the rate of complications.

Inevitably, there were several limitations in our study that should be pointed out. First, we only obtained study data within 6 months of follow-up, and the results of quality of life and changes in KAP beyond 6 months still need further exploration. Therefore, the mid- and long-term outcomes should be further explored in future studies with a longer follow-up. In addition, our study was conducted using a small-scale sample size, with all participants coming from Hangzhou District. This will inevitably lead to some statistical and regional biases in our findings, which to some extent limit the generalizability of our findings across all populations and different regions. Moreover, despite a random number table used in this study, the results of this study are not derived from the triple-blind design. The main reason may be that patients have the right to know the content of this project and decide whether to participate in this study. Finally, some potential unmeasured variables, such as type of insurance, patient adherence, and subgroup differences among different age, gender, and personal income, were not analyzed in this study. This requires more rigorous design and subgroup analysis of potential influencing factors in the future, so as to obtain more detailed and objective results. All in all, in view of the shortcomings mentioned above, more studies should be performed to eliminate the effects of confounding bias and confirm our conclusion in well-designed multi-center randomized controlled trials.

## 5 Conclusion

In summary, the peer-led intervention can enhance patients’ understanding of healthy knowledge, improve healthy attitudes and practices, reduce the rate of stoma complications, and eventually promote quality of life of patients with permanent ostomy. These advantages enable peer-led intervention to be a favorable approach for bladder cancer patients after permanent ostomy.

## Data Availability

The raw data supporting the conclusions of this article will be made available by the authors, without undue reservation.
